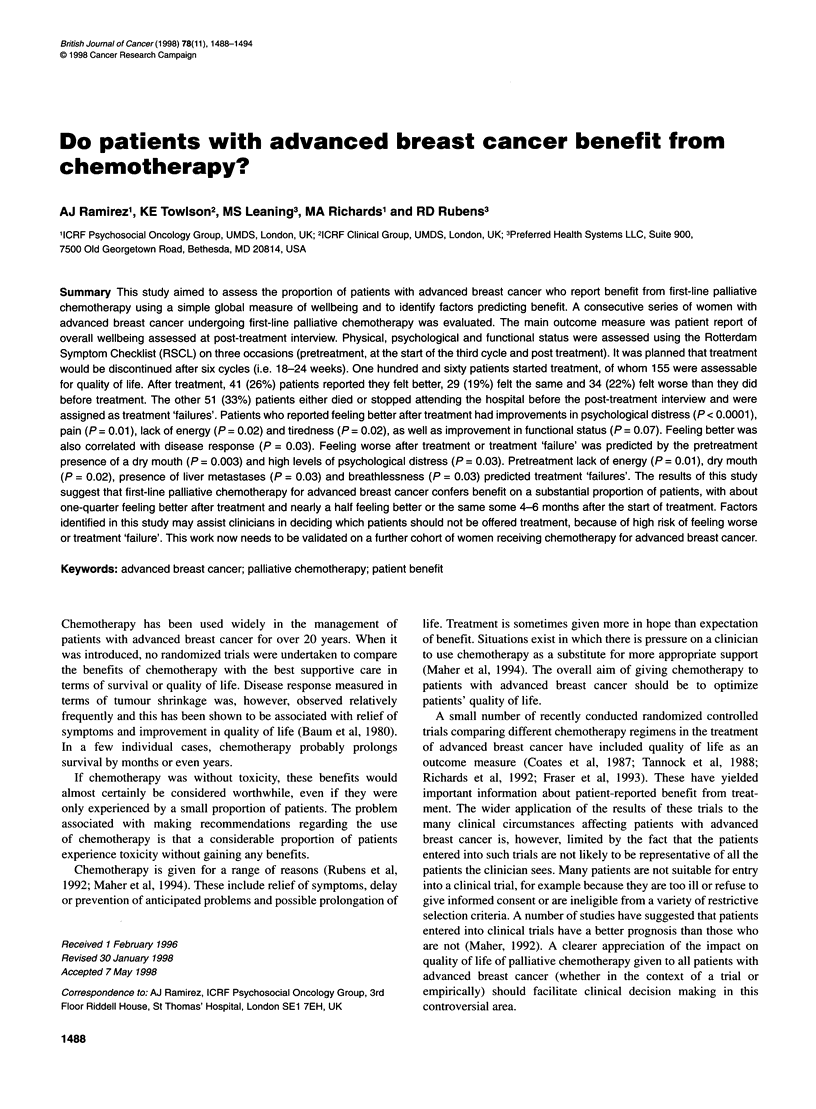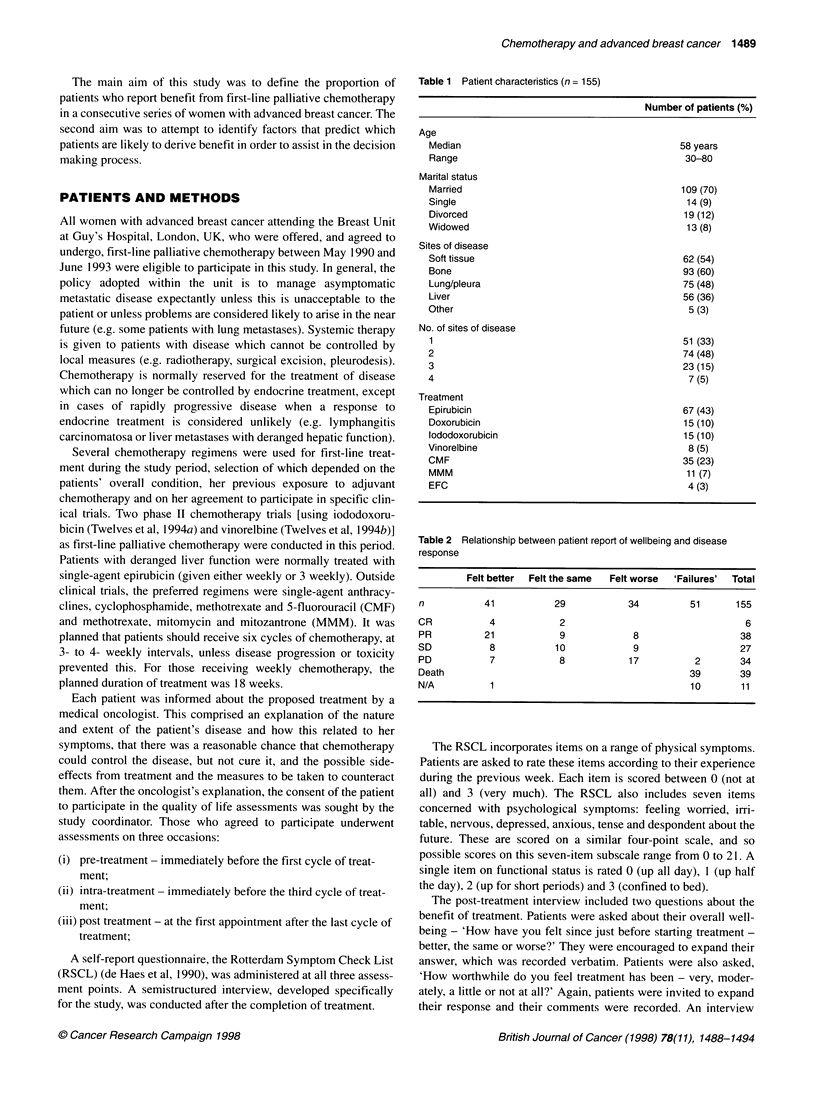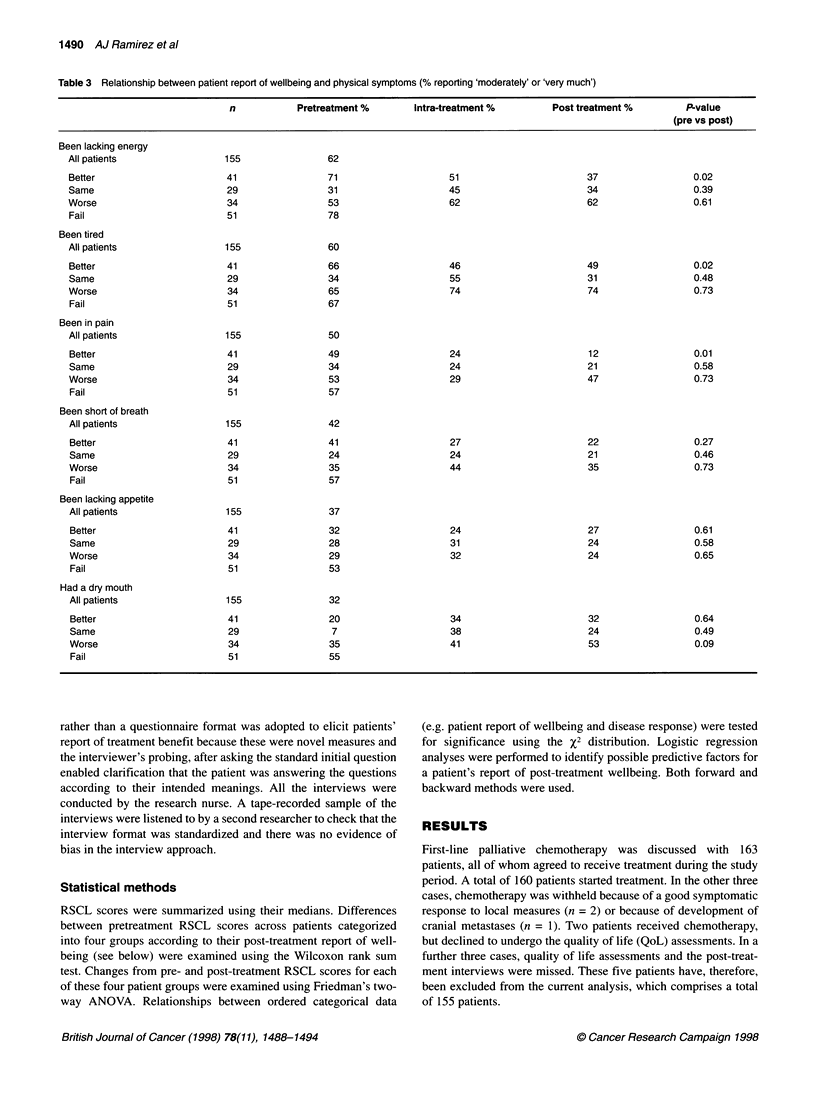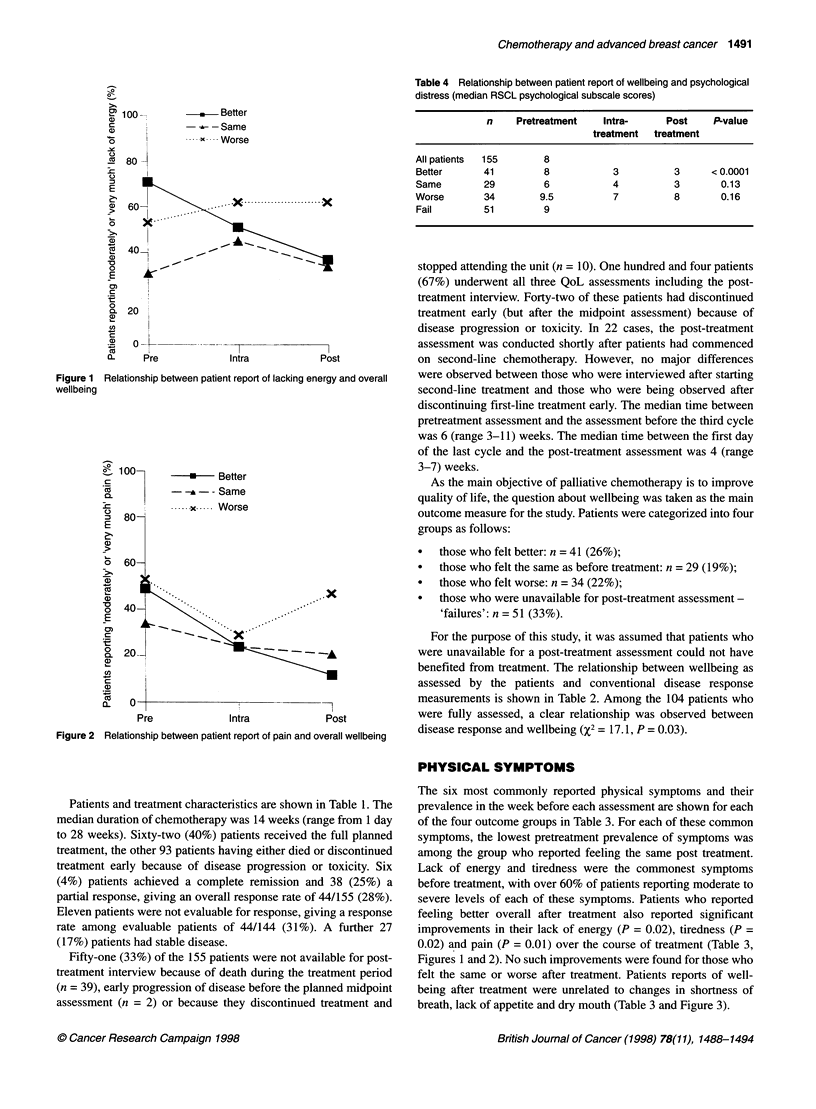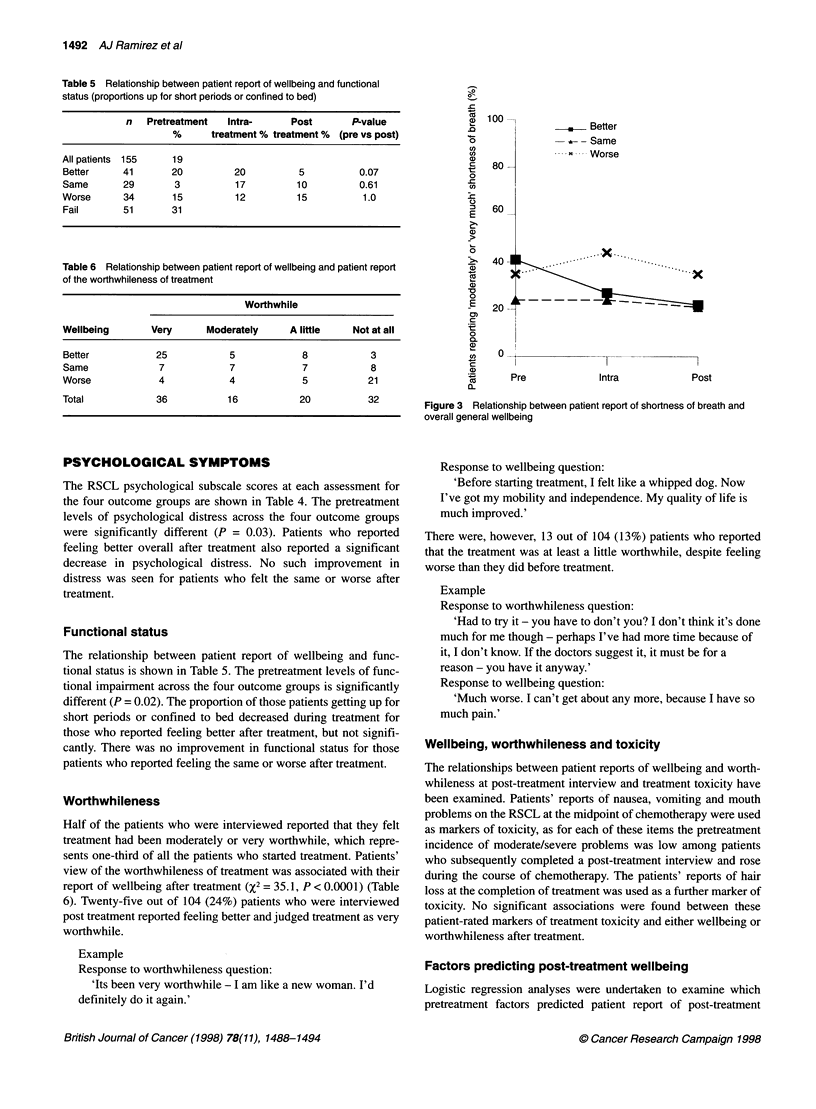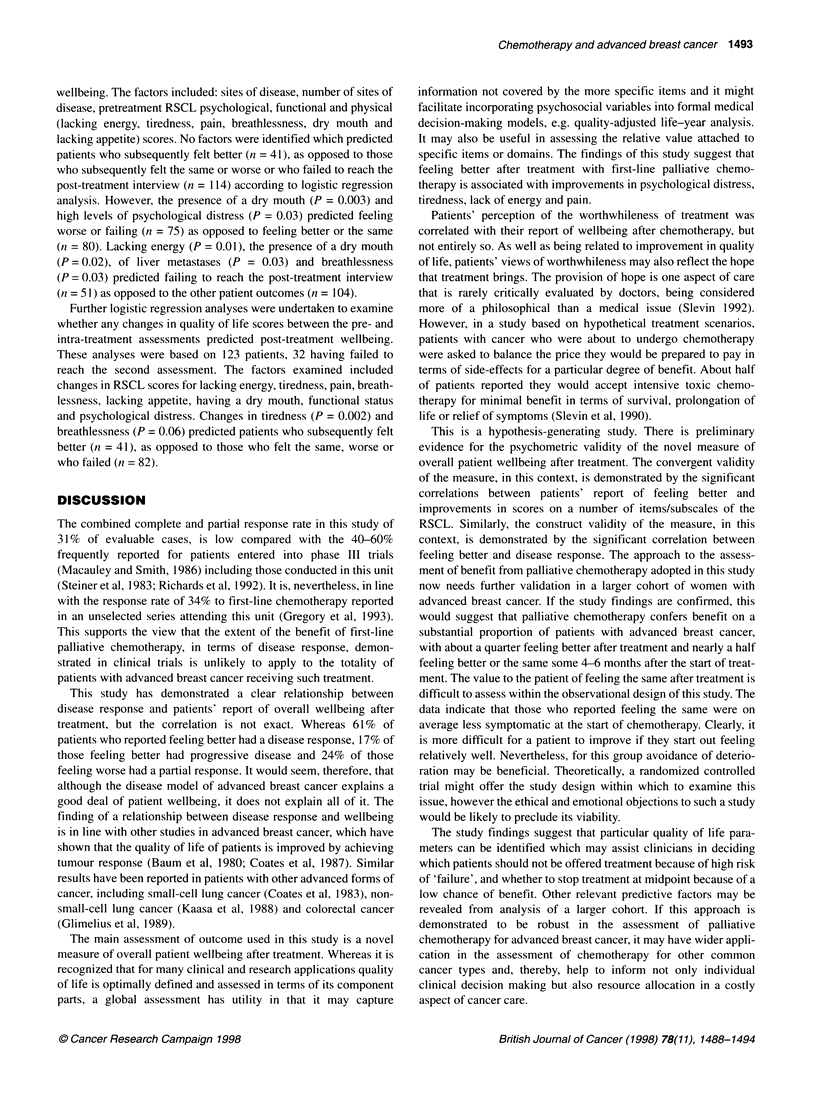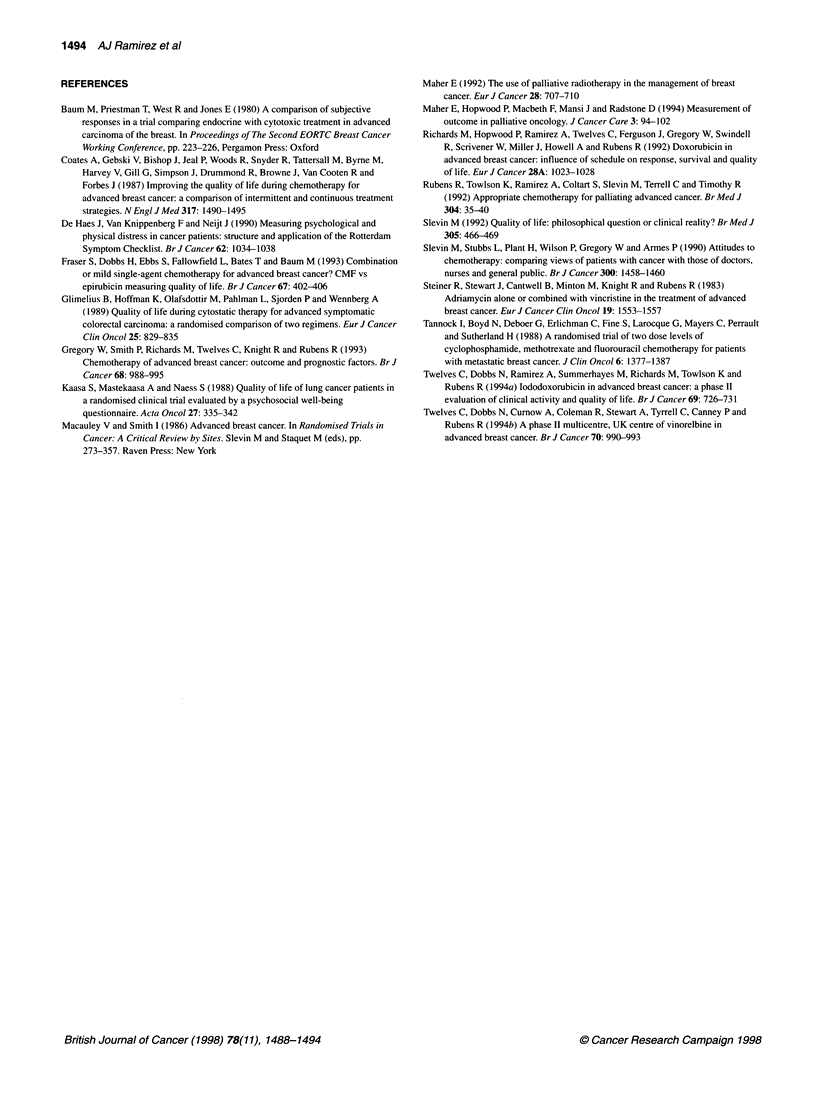# Do patients with advanced breast cancer benefit from chemotherapy?

**DOI:** 10.1038/bjc.1998.711

**Published:** 1998-12

**Authors:** A. J. Ramirez, K. E. Towlson, M. S. Leaning, M. A. Richards, R. D. Rubens

**Affiliations:** ICRF Psychosocial Oncology Group, UMDS, London, UK.

## Abstract

This study aimed to assess the proportion of patients with advanced breast cancer who report benefit from first-line palliative chemotherapy using a simple global measure of wellbeing and to identify factors predicting benefit. A consecutive series of women with advanced breast cancer undergoing first-line palliative chemotherapy was evaluated. The main outcome measure was patient report of overall wellbeing assessed at post-treatment interview. Physical, psychological and functional status were assessed using the Rotterdam Symptom Checklist (RSCL) on three occasions (pretreatment, at the start of the third cycle and post treatment). It was planned that treatment would be discontinued after six cycles (i.e. 18-24 weeks). One hundred and sixty patients started treatment, of whom 155 were assessable for quality of life. After treatment, 41 (26%) patients reported they felt better, 29 (19%) felt the same and 34 (22%) felt worse than they did before treatment. The other 51 (33%) patients either died or stopped attending the hospital before the post-treatment interview and were assigned as treatment 'failures'. Patients who reported feeling better after treatment had improvements in psychological distress (P < 0.0001), pain (P = 0.01), lack of energy (P = 0.02) and tiredness (P = 0.02), as well as improvement in functional status (P = 0.07). Feeling better was also correlated with disease response (P = 0.03). Feeling worse after treatment or treatment 'failure' was predicted by the pretreatment presence of a dry mouth (P = 0.003) and high levels of psychological distress (P = 0.03). Pretreatment lack of energy (P = 0.01), dry mouth (P = 0.02), presence of liver metastases (P = 0.03) and breathlessness (P = 0.03) predicted treatment 'failures'. The results of this study suggest that first-line palliative chemotherapy for advanced breast cancer confers benefit on a substantial proportion of patients, with about one-quarter feeling better after treatment and nearly a half feeling better or the same some 4-6 months after the start of treatment. Factors identified in this study may assist clinicians in deciding which patients should not be offered treatment, because of high risk of feeling worse or treatment 'failure'. This work now needs to be validated on a further cohort of women receiving chemotherapy for advanced breast cancer.


British Journal of Cancer (1998) 78(11), 1488–1494
© 1998 Cancer Research Campaign

### Do patients with advanced breast cancer benefit from chemotherapy?

**AJ Ramirez[1], KE Towlson[2], MS Leaning[3], MA Richards[1] and RD Rubens[3]**

[1]ICRF Psychosocial Oncology Group, UMDS, London, UK; [2]ICRF Clinical Group, UMDS, London, UK; [3]Preferred Health Systems LLC, Suite 900, 7500 Old Georgetown Road, Bethesda, MD 20814, USA

**Summary** This study aimed to assess the proportion of patients with advanced breast cancer who report benefit from first-line palliative chemotherapy using a simple global measure of wellbeing and to identify factors predicting benefit. A consecutive series of women with advanced breast cancer undergoing first-line palliative chemotherapy was evaluated. The main outcome measure was patient report of overall wellbeing assessed at post-treatment interview. Physical, psychological and functional status were assessed using the Rotterdam Symptom Checklist (RSCL) on three occasions (pretreatment, at the start of the third cycle and post treatment). It was planned that treatment would be discontinued after six cycles (i.e. 18–24 weeks). One hundred and sixty patients started treatment, of whom 155 were assessable for quality of life. After treatment, 41 (26%) patients reported they felt better, 29 (19%) felt the same and 34 (22%) felt worse than they did before treatment. The other 51 (33%) patients either died or stopped attending the hospital before the post-treatment interview and were assigned as treatment 'failures'. Patients who reported feeling better after treatment had improvements in psychological distress ($P < 0.0001$), pain ($P = 0.01$), lack of energy ($P = 0.02$) and tiredness ($P = 0.02$), as well as improvement in functional status ($P = 0.07$). Feeling better was also correlated with disease response ($P = 0.03$). Feeling worse after treatment or treatment 'failure' was predicted by the pretreatment presence of a dry mouth ($P = 0.003$) and high levels of psychological distress ($P = 0.03$). Pretreatment lack of energy ($P = 0.01$), dry mouth ($P = 0.02$), presence of liver metastases ($P = 0.03$) and breathlessness ($P = 0.03$) predicted treatment 'failures'. The results of this study suggest that first-line palliative chemotherapy for advanced breast cancer confers benefit on a substantial proportion of patients, with about one-quarter feeling better after treatment and nearly a half feeling better or the same some 4–6 months after the start of treatment. Factors identified in this study may assist clinicians in deciding which patients should not be offered treatment, because of high risk of feeling worse or treatment 'failure'. This work now needs to be validated on a further cohort of women receiving chemotherapy for advanced breast cancer.

**Keywords:** advanced breast cancer; palliative chemotherapy; patient benefit

Chemotherapy has been used widely in the management of patients with advanced breast cancer for over 20 years. When it was introduced, no randomized trials were undertaken to compare the benefits of chemotherapy with the best supportive care in terms of survival or quality of life. Disease response measured in terms of tumour shrinkage was, however, observed relatively frequently and this has been shown to be associated with relief of symptoms and improvement in quality of life (Baum et al, 1980). In a few individual cases, chemotherapy probably prolongs survival by months or even years.

If chemotherapy was without toxicity, these benefits would almost certainly be considered worthwhile, even if they were only experienced by a small proportion of patients. The problem associated with making recommendations regarding the use of chemotherapy is that a considerable proportion of patients experience toxicity without gaining any benefits.

Chemotherapy is given for a range of reasons (Rubens et al, 1992; Maher et al, 1994). These include relief of symptoms, delay or prevention of anticipated problems and possible prolongation of life. Treatment is sometimes given more in hope than expectation of benefit. Situations exist in which there is pressure on a clinician to use chemotherapy as a substitute for more appropriate support (Maher et al, 1994). The overall aim of giving chemotherapy to patients with advanced breast cancer should be to optimize patients' quality of life.

A small number of recently conducted randomized controlled trials comparing different chemotherapy regimens in the treatment of advanced breast cancer have included quality of life as an outcome measure (Coates et al, 1987; Tannock et al, 1988; Richards et al, 1992; Fraser et al, 1993). These have yielded important information about patient-reported benefit from treatment. The wider application of the results of these trials to the many clinical circumstances affecting patients with advanced breast cancer is, however, limited by the fact that the patients entered into such trials are not likely to be representative of all the patients the clinician sees. Many patients are not suitable for entry into a clinical trial, for example because they are too ill or refuse to give informed consent or are ineligible from a variety of restrictive selection criteria. A number of studies have suggested that patients entered into clinical trials have a better prognosis than those who are not (Maher, 1992). A clearer appreciation of the impact on quality of life of palliative chemotherapy given to all patients with advanced breast cancer (whether in the context of a trial or empirically) should facilitate clinical decision making in this controversial area.

*Received 1 February 1996*
*Revised 30 January 1998*
*Accepted 7 May 1998*

*Correspondence to:* AJ Ramirez, ICRF Psychosocial Oncology Group, 3rd Floor Riddell House, St Thomas' Hospital, London SE1 7EH, UK

The main aim of this study was to define the proportion of patients who report benefit from first-line palliative chemotherapy in a consecutive series of women with advanced breast cancer. The second aim was to attempt to identify factors that predict which patients are likely to derive benefit in order to assist in the decision making process.

### PATIENTS AND METHODS

All women with advanced breast cancer attending the Breast Unit at Guy's Hospital, London, UK, who were offered, and agreed to undergo, first-line palliative chemotherapy between May 1990 and June 1993 were eligible to participate in this study. In general, the policy adopted within the unit is to manage asymptomatic metastatic disease expectantly unless this is unacceptable to the patient or unless problems are considered likely to arise in the near future (e.g. some patients with lung metastases). Systemic therapy is given to patients with disease which cannot be controlled by local measures (e.g. radiotherapy, surgical excision, pleurodesis). Chemotherapy is normally reserved for the treatment of disease which can no longer be controlled by endocrine treatment, except in cases of rapidly progressive disease when a response to endocrine treatment is considered unlikely (e.g. lymphangitis carcinomatosa or liver metastases with deranged hepatic function).

Several chemotherapy regimens were used for first-line treatment during the study period, selection of which depended on the patients' overall condition, her previous exposure to adjuvant chemotherapy and on her agreement to participate in specific clinical trials. Two phase II chemotherapy trials [using iododoxorubicin (Twelves et al, 1994*a*) and vinorelbine (Twelves et al, 1994*b*)] as first-line palliative chemotherapy were conducted in this period. Patients with deranged liver function were normally treated with single-agent epirubicin (given either weekly or 3 weekly). Outside clinical trials, the preferred regimens were single-agent anthracyclines, cyclophosphamide, methotrexate and 5-fluorouracil (CMF) and methotrexate, mitomycin and mitozantrone (MMM). It was planned that patients should receive six cycles of chemotherapy, at 3- to 4- weekly intervals, unless disease progression or toxicity prevented this. For those receiving weekly chemotherapy, the planned duration of treatment was 18 weeks.

Each patient was informed about the proposed treatment by a medical oncologist. This comprised an explanation of the nature and extent of the patient's disease and how this related to her symptoms, that there was a reasonable chance that chemotherapy could control the disease, but not cure it, and the possible side-effects from treatment and the measures to be taken to counteract them. After the oncologist's explanation, the consent of the patient to participate in the quality of life assessments was sought by the study coordinator. Those who agreed to participate underwent assessments on three occasions:

(i) pre-treatment – immediately before the first cycle of treatment;
(ii) intra-treatment – immediately before the third cycle of treatment;
(iii) post treatment – at the first appointment after the last cycle of treatment;

A self-report questionnaire, the Rotterdam Symptom Check List (RSCL) (de Haes et al, 1990), was administered at all three assessment points. A semistructured interview, developed specifically for the study, was conducted after the completion of treatment.

**Table 1** Patient characteristics ($n = 155$)

| | Number of patients (%) |
|---|---|
| Age | |
| Median | 58 years |
| Range | 30–80 |
| Marital status | |
| Married | 109 (70) |
| Single | 14 (9) |
| Divorced | 19 (12) |
| Widowed | 13 (8) |
| Sites of disease | |
| Soft tissue | 62 (54) |
| Bone | 93 (60) |
| Lung/pleura | 75 (48) |
| Liver | 56 (36) |
| Other | 5 (3) |
| No. of sites of disease | |
| 1 | 51 (33) |
| 2 | 74 (48) |
| 3 | 23 (15) |
| 4 | 7 (5) |
| Treatment | |
| Epirubicin | 67 (43) |
| Doxorubicin | 15 (10) |
| Iododoxorubicin | 15 (10) |
| Vinorelbine | 8 (5) |
| CMF | 35 (23) |
| MMM | 11 (7) |
| EFC | 4 (3) |

**Table 2** Relationship between patient report of wellbeing and disease response

| | Felt better | Felt the same | Felt worse | 'Failures' | Total |
|---|---|---|---|---|---|
| *n* | 41 | 29 | 34 | 51 | 155 |
| CR | 4 | 2 | | | 6 |
| PR | 21 | 9 | 8 | | 38 |
| SD | 8 | 10 | 9 | | 27 |
| PD | 7 | 8 | 17 | 2 | 34 |
| Death | | | | 39 | 39 |
| N/A | 1 | | | 10 | 11 |

The RSCL incorporates items on a range of physical symptoms. Patients are asked to rate these items according to their experience during the previous week. Each item is scored between 0 (not at all) and 3 (very much). The RSCL also includes seven items concerned with psychological symptoms: feeling worried, irritable, nervous, depressed, anxious, tense and despondent about the future. These are scored on a similar four-point scale, and so possible scores on this seven-item subscale range from 0 to 21. A single item on functional status is rated 0 (up all day), 1 (up half the day), 2 (up for short periods) and 3 (confined to bed).

The post-treatment interview included two questions about the benefit of treatment. Patients were asked about their overall wellbeing – 'How have you felt since just before starting treatment – better, the same or worse?' They were encouraged to expand their answer, which was recorded verbatim. Patients were also asked, 'How worthwhile do you feel treatment has been – very, moderately, a little or not at all?' Again, patients were invited to expand their response and their comments were recorded. An interview

© Cancer Research Campaign 1998

**Table 3** Relationship between patient report of wellbeing and physical symptoms (% reporting 'moderately' or 'very much')

| | n | Pretreatment % | Intra-treatment % | Post treatment % | P-value (pre vs post) |
|---|---|---|---|---|---|
| Been lacking energy | | | | | |
| All patients | 155 | 62 | | | |
| Better | 41 | 71 | 51 | 37 | 0.02 |
| Same | 29 | 31 | 45 | 34 | 0.39 |
| Worse | 34 | 53 | 62 | 62 | 0.61 |
| Fail | 51 | 78 | | | |
| Been tired | | | | | |
| All patients | 155 | 60 | | | |
| Better | 41 | 66 | 46 | 49 | 0.02 |
| Same | 29 | 34 | 55 | 31 | 0.48 |
| Worse | 34 | 65 | 74 | 74 | 0.73 |
| Fail | 51 | 67 | | | |
| Been in pain | | | | | |
| All patients | 155 | 50 | | | |
| Better | 41 | 49 | 24 | 12 | 0.01 |
| Same | 29 | 34 | 24 | 21 | 0.58 |
| Worse | 34 | 53 | 29 | 47 | 0.73 |
| Fail | 51 | 57 | | | |
| Been short of breath | | | | | |
| All patients | 155 | 42 | | | |
| Better | 41 | 41 | 27 | 22 | 0.27 |
| Same | 29 | 24 | 24 | 21 | 0.46 |
| Worse | 34 | 35 | 44 | 35 | 0.73 |
| Fail | 51 | 57 | | | |
| Been lacking appetite | | | | | |
| All patients | 155 | 37 | | | |
| Better | 41 | 32 | 24 | 27 | 0.61 |
| Same | 29 | 28 | 31 | 24 | 0.58 |
| Worse | 34 | 29 | 32 | 24 | 0.65 |
| Fail | 51 | 53 | | | |
| Had a dry mouth | | | | | |
| All patients | 155 | 32 | | | |
| Better | 41 | 20 | 34 | 32 | 0.64 |
| Same | 29 | 7 | 38 | 24 | 0.49 |
| Worse | 34 | 35 | 41 | 53 | 0.09 |
| Fail | 51 | 55 | | | |

rather than a questionnaire format was adopted to elicit patients' report of treatment benefit because these were novel measures and the interviewer's probing, after asking the standard initial question enabled clarification that the patient was answering the questions according to their intended meanings. All the interviews were conducted by the research nurse. A tape-recorded sample of the interviews were listened to by a second researcher to check that the interview format was standardized and there was no evidence of bias in the interview approach.

### Statistical methods

RSCL scores were summarized using their medians. Differences between pretreatment RSCL scores across patients categorized into four groups according to their post-treatment report of wellbeing (see below) were examined using the Wilcoxon rank sum test. Changes from pre- and post-treatment RSCL scores for each of these four patient groups were examined using Friedman's two-way ANOVA. Relationships between ordered categorical data (e.g. patient report of wellbeing and disease response) were tested for significance using the $\chi^2$ distribution. Logistic regression analyses were performed to identify possible predictive factors for a patient's report of post-treatment wellbeing. Both forward and backward methods were used.

### RESULTS

First-line palliative chemotherapy was discussed with 163 patients, all of whom agreed to receive treatment during the study period. A total of 160 patients started treatment. In the other three cases, chemotherapy was withheld because of a good symptomatic response to local measures ($n$ = 2) or because of development of cranial metastases ($n$ = 1). Two patients received chemotherapy, but declined to undergo the quality of life (QoL) assessments. In a further three cases, quality of life assessments and the post-treatment interviews were missed. These five patients have, therefore, been excluded from the current analysis, which comprises a total of 155 patients.

© Cancer Research Campaign 1998

**Figure 1** Relationship between patient report of lacking energy and overall wellbeing

**Figure 2** Relationship between patient report of pain and overall wellbeing

Patients and treatment characteristics are shown in Table 1. The median duration of chemotherapy was 14 weeks (range from 1 day to 28 weeks). Sixty-two (40%) patients received the full planned treatment, the other 93 patients having either died or discontinued treatment early because of disease progression or toxicity. Six (4%) patients achieved a complete remission and 38 (25%) a partial response, giving an overall response rate of 44/155 (28%). Eleven patients were not evaluable for response, giving a response rate among evaluable patients of 44/144 (31%). A further 27 (17%) patients had stable disease.

Fifty-one (33%) of the 155 patients were not available for post-treatment interview because of death during the treatment period ($n$ = 39), early progression of disease before the planned midpoint assessment ($n$ = 2) or because they discontinued treatment and stopped attending the unit ($n$ = 10). One hundred and four patients (67%) underwent all three QoL assessments including the post-treatment interview. Forty-two of these patients had discontinued treatment early (but after the midpoint assessment) because of disease progression or toxicity. In 22 cases, the post-treatment assessment was conducted shortly after patients had commenced on second-line chemotherapy. However, no major differences were observed between those who were interviewed after starting second-line treatment and those who were being observed after discontinuing first-line treatment early. The median time between pretreatment assessment and the assessment before the third cycle was 6 (range 3–11) weeks. The median time between the first day of the last cycle and the post-treatment assessment was 4 (range 3–7) weeks.

**Table 4** Relationship between patient report of wellbeing and psychological distress (median RSCL psychological subscale scores)

| | *n* | Pretreatment | Intra-treatment | Post treatment | *P*-value |
|---|---|---|---|---|---|
| All patients | 155 | 8 | | | |
| Better | 41 | 8 | 3 | 3 | < 0.0001 |
| Same | 29 | 6 | 4 | 3 | 0.13 |
| Worse | 34 | 9.5 | 7 | 8 | 0.16 |
| Fail | 51 | 9 | | | |

As the main objective of palliative chemotherapy is to improve quality of life, the question about wellbeing was taken as the main outcome measure for the study. Patients were categorized into four groups as follows:

- those who felt better: $n$ = 41 (26%);
- those who felt the same as before treatment: $n$ = 29 (19%);
- those who felt worse: $n$ = 34 (22%);
- those who were unavailable for post-treatment assessment – 'failures': $n$ = 51 (33%).

For the purpose of this study, it was assumed that patients who were unavailable for a post-treatment assessment could not have benefited from treatment. The relationship between wellbeing as assessed by the patients and conventional disease response measurements is shown in Table 2. Among the 104 patients who were fully assessed, a clear relationship was observed between disease response and wellbeing ($\chi^2$ = 17.1, $P$ = 0.03).

### PHYSICAL SYMPTOMS

The six most commonly reported physical symptoms and their prevalence in the week before each assessment are shown for each of the four outcome groups in Table 3. For each of these common symptoms, the lowest pretreatment prevalence of symptoms was among the group who reported feeling the same post treatment. Lack of energy and tiredness were the commonest symptoms before treatment, with over 60% of patients reporting moderate to severe levels of each of these symptoms. Patients who reported feeling better overall after treatment also reported significant improvements in their lack of energy ($P$ = 0.02), tiredness ($P$ = 0.02) and pain ($P$ = 0.01) over the course of treatment (Table 3, Figures 1 and 2). No such improvements were found for those who felt the same or worse after treatment. Patients reports of well-being after treatment were unrelated to changes in shortness of breath, lack of appetite and dry mouth (Table 3 and Figure 3).

© Cancer Research Campaign 1998 

Table 5 Relationship between patient report of wellbeing and functional status (proportions up for short periods or confined to bed)

| | *n* | Pretreatment % | Intra-treatment % | Post treatment % | *P*-value (pre vs post) |
|---|---|---|---|---|---|
| All patients | 155 | 19 | | | |
| Better | 41 | 20 | 20 | 5 | 0.07 |
| Same | 29 | 3 | 17 | 10 | 0.61 |
| Worse | 34 | 15 | 12 | 15 | 1.0 |
| Fail | 51 | 31 | | | |

Table 6 Relationship between patient report of wellbeing and patient report of the worthwhileness of treatment

| | Worthwhile | | | |
|---|---|---|---|---|
| Wellbeing | Very | Moderately | A little | Not at all |
| Better | 25 | 5 | 8 | 3 |
| Same | 7 | 7 | 7 | 8 |
| Worse | 4 | 4 | 5 | 21 |
| Total | 36 | 16 | 20 | 32 |

Figure 3 Relationship between patient report of shortness of breath and overall general wellbeing

### PSYCHOLOGICAL SYMPTOMS

The RSCL psychological subscale scores at each assessment for the four outcome groups are shown in Table 4. The pretreatment levels of psychological distress across the four outcome groups were significantly different ($P = 0.03$). Patients who reported feeling better overall after treatment also reported a significant decrease in psychological distress. No such improvement in distress was seen for patients who felt the same or worse after treatment.

### Functional status

The relationship between patient report of wellbeing and functional status is shown in Table 5. The pretreatment levels of functional impairment across the four outcome groups is significantly different ($P = 0.02$). The proportion of those patients getting up for short periods or confined to bed decreased during treatment for those who reported feeling better after treatment, but not significantly. There was no improvement in functional status for those patients who reported feeling the same or worse after treatment.

### Worthwhileness

Half of the patients who were interviewed reported that they felt treatment had been moderately or very worthwhile, which represents one-third of all the patients who started treatment. Patients' view of the worthwhileness of treatment was associated with their report of wellbeing after treatment ($\chi^2 = 35.1$, $P < 0.0001$) (Table 6). Twenty-five out of 104 (24%) patients who were interviewed post treatment reported feeling better and judged treatment as very worthwhile.

Example
Response to worthwhileness question:
'Its been very worthwhile – I am like a new woman. I'd definitely do it again.'
Response to wellbeing question:
'Before starting treatment, I felt like a whipped dog. Now I've got my mobility and independence. My quality of life is much improved.'

There were, however, 13 out of 104 (13%) patients who reported that the treatment was at least a little worthwhile, despite feeling worse than they did before treatment.

Example
Response to worthwhileness question:
'Had to try it – you have to don't you? I don't think it's done much for me though – perhaps I've had more time because of it, I don't know. If the doctors suggest it, it must be for a reason – you have it anyway.'
Response to wellbeing question:
'Much worse. I can't get about any more, because I have so much pain.'

### Wellbeing, worthwhileness and toxicity

The relationships between patient reports of wellbeing and worthwhileness at post-treatment interview and treatment toxicity have been examined. Patients' reports of nausea, vomiting and mouth problems on the RSCL at the midpoint of chemotherapy were used as markers of toxicity, as for each of these items the pretreatment incidence of moderate/severe problems was low among patients who subsequently completed a post-treatment interview and rose during the course of chemotherapy. The patients' reports of hair loss at the completion of treatment was used as a further marker of toxicity. No significant associations were found between these patient-rated markers of treatment toxicity and either wellbeing or worthwhileness after treatment.

### Factors predicting post-treatment wellbeing

Logistic regression analyses were undertaken to examine which pretreatment factors predicted patient report of post-treatment

© Cancer Research Campaign 1998

wellbeing. The factors included: sites of disease, number of sites of disease, pretreatment RSCL psychological, functional and physical (lacking energy, tiredness, pain, breathlessness, dry mouth and lacking appetite) scores. No factors were identified which predicted patients who subsequently felt better ($n = 41$), as opposed to those who subsequently felt the same or worse or who failed to reach the post-treatment interview ($n = 114$) according to logistic regression analysis. However, the presence of a dry mouth ($P = 0.003$) and high levels of psychological distress ($P = 0.03$) predicted feeling worse or failing ($n = 75$) as opposed to feeling better or the same ($n = 80$). Lacking energy ($P = 0.01$), the presence of a dry mouth ($P = 0.02$), of liver metastases ($P = 0.03$) and breathlessness ($P = 0.03$) predicted failing to reach the post-treatment interview ($n = 51$) as opposed to the other patient outcomes ($n = 104$).

Further logistic regression analyses were undertaken to examine whether any changes in quality of life scores between the pre- and intra-treatment assessments predicted post-treatment wellbeing. These analyses were based on 123 patients, 32 having failed to reach the second assessment. The factors examined included changes in RSCL scores for lacking energy, tiredness, pain, breathlessness, lacking appetite, having a dry mouth, functional status and psychological distress. Changes in tiredness ($P = 0.002$) and breathlessness ($P = 0.06$) predicted patients who subsequently felt better ($n = 41$), as opposed to those who felt the same, worse or who failed ($n = 82$).

### DISCUSSION

The combined complete and partial response rate in this study of 31% of evaluable cases, is low compared with the 40–60% frequently reported for patients entered into phase III trials (Macauley and Smith, 1986) including those conducted in this unit (Steiner et al, 1983; Richards et al, 1992). It is, nevertheless, in line with the response rate of 34% to first-line chemotherapy reported in an unselected series attending this unit (Gregory et al, 1993). This supports the view that the extent of the benefit of first-line palliative chemotherapy, in terms of disease response, demonstrated in clinical trials is unlikely to apply to the totality of patients with advanced breast cancer receiving such treatment.

This study has demonstrated a clear relationship between disease response and patients' report of overall wellbeing after treatment, but the correlation is not exact. Whereas 61% of patients who reported feeling better had a disease response, 17% of those feeling better had progressive disease and 24% of those feeling worse had a partial response. It would seem, therefore, that although the disease model of advanced breast cancer explains a good deal of patient wellbeing, it does not explain all of it. The finding of a relationship between disease response and wellbeing is in line with other studies in advanced breast cancer, which have shown that the quality of life of patients is improved by achieving tumour response (Baum et al, 1980; Coates et al, 1987). Similar results have been reported in patients with other advanced forms of cancer, including small-cell lung cancer (Coates et al, 1983), non-small-cell lung cancer (Kaasa et al, 1988) and colorectal cancer (Glimelius et al, 1989).

The main assessment of outcome used in this study is a novel measure of overall patient wellbeing after treatment. Whereas it is recognized that for many clinical and research applications quality of life is optimally defined and assessed in terms of its component parts, a global assessment has utility in that it may capture information not covered by the more specific items and it might facilitate incorporating psychosocial variables into formal medical decision-making models, e.g. quality-adjusted life–year analysis. It may also be useful in assessing the relative value attached to specific items or domains. The findings of this study suggest that feeling better after treatment with first-line palliative chemotherapy is associated with improvements in psychological distress, tiredness, lack of energy and pain.

Patients' perception of the worthwhileness of treatment was correlated with their report of wellbeing after chemotherapy, but not entirely so. As well as being related to improvement in quality of life, patients' views of worthwhileness may also reflect the hope that treatment brings. The provision of hope is one aspect of care that is rarely critically evaluated by doctors, being considered more of a philosophical than a medical issue (Slevin 1992). However, in a study based on hypothetical treatment scenarios, patients with cancer who were about to undergo chemotherapy were asked to balance the price they would be prepared to pay in terms of side-effects for a particular degree of benefit. About half of patients reported they would accept intensive toxic chemotherapy for minimal benefit in terms of survival, prolongation of life or relief of symptoms (Slevin et al, 1990).

This is a hypothesis-generating study. There is preliminary evidence for the psychometric validity of the novel measure of overall patient wellbeing after treatment. The convergent validity of the measure, in this context, is demonstrated by the significant correlations between patients' report of feeling better and improvements in scores on a number of items/subscales of the RSCL. Similarly, the construct validity of the measure, in this context, is demonstrated by the significant correlation between feeling better and disease response. The approach to the assessment of benefit from palliative chemotherapy adopted in this study now needs further validation in a larger cohort of women with advanced breast cancer. If the study findings are confirmed, this would suggest that palliative chemotherapy confers benefit on a substantial proportion of patients with advanced breast cancer, with about a quarter feeling better after treatment and nearly a half feeling better or the same some 4–6 months after the start of treatment. The value to the patient of feeling the same after treatment is difficult to assess within the observational design of this study. The data indicate that those who reported feeling the same were on average less symptomatic at the start of chemotherapy. Clearly, it is more difficult for a patient to improve if they start out feeling relatively well. Nevertheless, for this group avoidance of deterioration may be beneficial. Theoretically, a randomized controlled trial might offer the study design within which to examine this issue, however the ethical and emotional objections to such a study would be likely to preclude its viability.

The study findings suggest that particular quality of life parameters can be identified which may assist clinicians in deciding which patients should not be offered treatment because of high risk of 'failure', and whether to stop treatment at midpoint because of a low chance of benefit. Other relevant predictive factors may be revealed from analysis of a larger cohort. If this approach is demonstrated to be robust in the assessment of palliative chemotherapy for advanced breast cancer, it may have wider application in the assessment of chemotherapy for other common cancer types and, thereby, help to inform not only individual clinical decision making but also resource allocation in a costly aspect of cancer care.

© Cancer Research Campaign 1998

### REFERENCES

Baum M, Priestman T, West R and Jones E (1980) A comparison of subjective responses in a trial comparing endocrine with cytotoxic treatment in advanced carcinoma of the breast. In *Proceedings of The Second EORTC Breast Cancer Working Conference*, pp. 223–226, Pergamon Press: Oxford

Coates A, Gebski V, Bishop J, Jeal P, Woods R, Snyder R, Tattersall M, Byrne M, Harvey V, Gill G, Simpson J, Drummond R, Browne J, Van Cooten R and Forbes J (1987) Improving the quality of life during chemotherapy for advanced breast cancer: a comparison of intermittent and continuous treatment strategies. *N Engl J Med* **317**: 1490–1495

De Haes J, Van Knippenberg F and Neijt J (1990) Measuring psychological and physical distress in cancer patients: structure and application of the Rotterdam Symptom Checklist. *Br J Cancer* **62**: 1034–1038

Fraser S, Dobbs H, Ebbs S, Fallowfield L, Bates T and Baum M (1993) Combination or mild single-agent chemotherapy for advanced breast cancer? CMF vs epirubicin measuring quality of life. *Br J Cancer* **67**: 402–406

Glimelius B, Hoffman K, Olafsdottir M, Pahlman L, Sjorden P and Wennberg A (1989) Quality of life during cytostatic therapy for advanced symptomatic colorectal carcinoma: a randomised comparison of two regimens. *Eur J Cancer Clin Oncol* **25**: 829–835

Gregory W, Smith P, Richards M, Twelves C, Knight R and Rubens R (1993) Chemotherapy of advanced breast cancer: outcome and prognostic factors. *Br J Cancer* **68**: 988–995

Kaasa S, Mastekaasa A and Naess S (1988) Quality of life of lung cancer patients in a randomised clinical trial evaluated by a psychosocial well-being questionnaire. *Acta Oncol* **27**: 335–342

Macauley V and Smith I (1986) Advanced breast cancer. In *Randomised Trials in Cancer: A Critical Review by Sites*. Slevin M and Staquet M (eds), pp. 273–357. Raven Press: New York

Maher E (1992) The use of palliative radiotherapy in the management of breast cancer. *Eur J Cancer* **28**: 707–710

Maher E, Hopwood P, Macbeth F, Mansi J and Radstone D (1994) Measurement of outcome in palliative oncology. *J Cancer Care* **3**: 94–102

Richards M, Hopwood P, Ramirez A, Twelves C, Ferguson J, Gregory W, Swindell R, Scrivener W, Miller J, Howell A and Rubens R (1992) Doxorubicin in advanced breast cancer: influence of schedule on response, survival and quality of life. *Eur J Cancer* **28A**: 1023–1028

Rubens R, Towlson K, Ramirez A, Coltart S, Slevin M, Terrell C and Timothy R (1992) Appropriate chemotherapy for palliating advanced cancer. *Br Med J* **304**: 35–40

Slevin M (1992) Quality of life: philosophical question or clinical reality? *Br Med J* **305**: 466–469

Slevin M, Stubbs L, Plant H, Wilson P, Gregory W and Armes P (1990) Attitudes to chemotherapy: comparing views of patients with cancer with those of doctors, nurses and general public. *Br J Cancer* **300**: 1458–1460

Steiner R, Stewart J, Cantwell B, Minton M, Knight R and Rubens R (1983) Adriamycin alone or combined with vincristine in the treatment of advanced breast cancer. *Eur J Cancer Clin Oncol* **19**: 1553–1557

Tannock I, Boyd N, Deboer G, Erlichman C, Fine S, Larocque G, Mayers C, Perrault and Sutherland H (1988) A randomised trial of two dose levels of cyclophosphamide, methotrexate and fluorouracil chemotherapy for patients with metastatic breast cancer. *J Clin Oncol* **6**: 1377–1387

Twelves C, Dobbs N, Ramirez A, Summerhayes M, Richards M, Towlson K and Rubens R (1994*a*) Iododoxorubicin in advanced breast cancer: a phase II evaluation of clinical activity and quality of life. *Br J Cancer* **69**: 726–731

Twelves C, Dobbs N, Curnow A, Coleman R, Stewart A, Tyrrell C, Canney P and Rubens R (1994*b*) A phase II multicentre, UK centre of vinorelbine in advanced breast cancer. *Br J Cancer* **70**: 990–993

*© Cancer Research Campaign 1998*